# Viruela símica: serie de casos

**DOI:** 10.31053/1853.0605.v80.n4.42303

**Published:** 2023-12-26

**Authors:** Victoria Micaela Pieretti, Marina Agriello, Mayled Maria Delgado Molina, Paula Bonaura, Camila Anabel Ramallo, Eugenia Miraglia, Florencia Dauria, María Victoria Garritano, María Roxana Maradeo

**Affiliations:** 1 Hospital Interzonal General de Agudos "José de San Martín" La Plata Buenos Aires Argentina

**Keywords:** Virus de la Viruela de los Monos, Viruela del Mono, Zoonosis Virales, Monkeypox virus, Monkeypox, Viral Zoonoses, Vírus da Varíola dos Macacos, Varíola dos Macacos, Zoonoses Virais

## Abstract

**Introducción:**

el brote reciente de mpox fue considerado una emergencia de salud pública internacional.

**Objetivo:**

describir las características epidemiológicas y clínicas de mpox en un hospital de la provincia de Buenos Aires.

**Métodos:**

estudio de serie de casos en pacientes ≥ 15 años en el servicio de Dermatología del Hospital Interzonal General de Agudos “San Martín” de La Plata entre agosto y noviembre del año 2022.

**Resultados:**

se incluyeron 10 pacientes. La edad media de presentación fue 35 años. Siete de los pacientes eran hombres y las tres restantes fueron mujeres. La mayoría de ellos presentó relación sexual de riesgo como antecedente epidemiológico. En el 70% de los pacientes se observaron pseudopústulas y todos tuvieron lesiones a nivel genital, glútea o perianal. Las complicaciones observadas fueron: edema local, proctitis, conjuntivis y faringitis.

**Conclusión:**

presentamos 3 pacientes de sexo femenino del total de 24 mujeres reportadas en el país, que representan sólo el 2% de las infecciones por mpox en Argentina. En la mayoría de los casos observamos pseudopústulas, lesión elemental descripta recientemente para esta entidad. Un paciente presentó compromiso ocular, complicación informada en un 1% de los casos en el brote actual.

## Abstract (en)

**Introduction:**

the recent mpox outbreak was considered an international public health emergency.

**Objective:**

describe the epidemiological and clinical characteristics of mpox in a hospital in the province of Buenos Aires.

**Methods:**

case series study in patients ≥15 years of age in the Dermatology service of the Hospital Interzonal General de Agudos "San Martín" in La Plata between August and November 2022.

**Results:**

10 patients were included. The mean age of presentation was 35 years. Seven of the patients were men and the remaining three were women. Most of them presented risky sexual intercourse as an epidemiological history. Pseudopustules were observed in 70% of the patients and all had genital, gluteal or perianal lesions. The complications observed were: local edema, proctitis, conjunctivitis and pharyngitis.

**Conclusion:**

we present 3 female patients out of a total of 24 women reported in the country, which represent only 2% of mpox infections in Argentina. In most cases we observe pseudopustules, an elementary lesion recently described for this entity. One patient presented ocular involvement, a complication reported in 1% of cases in the current outbreak.

## Resumo

**Introdução:**

O recente surto de mpox foi considerado uma emergência de saúde pública internacional.

**Objetivo:**

descrever as características epidemiológicas e clínicas do mpox em um hospital da província de Buenos Aires.

**Métodos:**

estudo de série de casos em pacientes ≥15 anos no serviço de Dermatologia do Hospital Interzonal General de Agudos "San Martín" em La Plata entre agosto e novembro de 2022.

**Resultados:**

10 pacientes foram incluídos. A idade média de apresentação foi de 35 anos. Sete dos pacientes eram homens e os três restantes eram mulheres. A maioria apresentou relação sexual de risco como histórico epidemiológico. Pseudopústulas foram observadas em 70% dos pacientes e todos apresentavam lesões genitais, glúteas ou perianais. As complicações observadas foram: edema local, proctite, conjuntivite e faringite.

**Conclusão:**

apresentamos 3 pacientes do sexo feminino de um total de 24 mulheres notificadas no país, que representam apenas 2% das infecções por mpox na Argentina. Na maioria dos casos observamos pseudopústulas, lesão elementar recentemente descrita para esta entidade. Um paciente apresentou envolvimento ocular, complicação relatada em 1% dos casos no atual surto.

**CONCEPTOS CLAVE**

Qué se sabe sobre el tema: El brote reciente de mpox fue considerado una emergencia de salud pública de importancia internacional hasta mayo del año 2023. Las manifestaciones cutáneas aparecieron principalmente en el área anogenital de hombres jóvenes que tenían sexo con hombres, pero la enfermedad puede afectar a cualquier persona con conductas sexuales de riesgo.

Qué aporta este trabajo: Conocer las manifestaciones clínicas de la enfermedad en pacientes de sexo masculino y femenino. Describir la experiencia de un hospital público interzonal de la provincia de Buenos Aires, Argentina, durante el brote de mpox.

**Divulgación**

Desde el año 1970 se describieron casos de viruela símica en países de África por contacto con animales infectados. En mayo del año 2022 aparecieron múltiples casos en otros continentes sin relación con animales. La mayoría de estas personas presentaba lesiones genitales por lo que se relacionó su contagio con conductas sexuales de riesgo. A nivel mundial se reportaron más de 87000 casos de esta infección. La mayoría fueron leves y de buen pronóstico.

## Introducción

La viruela símica (mpox) es una zoonosis viral endémica en países de África. Desde mayo del año 2022 se identificaron múltiples casos en países no endémicos que afectan principalmente a hombres que tienen sexo con hombres, dentro de los cuales se incluye Argentina. ^1-10^ El brote actual se caracteriza por manifestaciones atípicas y diferentes a la presentación tradicional. ^1 2^

El objetivo de este trabajo es describir las características epidemiológicas y clínicas de mpox en pacientes de ambos géneros en un hospital de la provincia de Buenos Aires.

## Materiales y Métodos

Se realizó un estudio de serie de casos en pacientes ≥ de 15 años de género masculino y femenino con diagnóstico de mpox mediante prueba de reacción en cadena de la polimerasa (PCR) en el servicio de dermatología del Hospital Interzonal General de Agudos "San Martín" de La Plata entre agosto y noviembre del año 2022.

Se recabó información acerca de edad, sexo, género, orientación sexual, diagnóstico previo de infección por el virus de la inmunodeficiencia humana (VIH), infecciones de transmisión sexual (ITS) concomitantes, antecedentes de viajes y relación sexual sin protección, síntomas sistémicos asociados, manifestaciones cutáneas y complicaciones.

## Resultados

Se incluyeron 10 historias clínicas de pacientes con diagnóstico de mpox oriundos de la provincia de Buenos Aires.

Las características epidemiológicas de los casos se detallan en la tabla 1. Siete de los pacientes eran hombres (5 de ellos homosexuales) y las tres restantes fueron mujeres heterosexuales. Ningún paciente era transgénero.

**Tabla 1:** Características demográficas y epidemiológicas de los diez casos diagnosticados en el Servicio de Dermatología del HIGA "Gral. San Martín" de La Plata.

	N = 10
CARACTERÍSTICAS	FRECUENCIA
Edad en años (mediana)	35 (20-51)
Sexo:	
Masculino	7
Femenino	3
Orientación sexual:	
Heterosexual	5
Homosexual	5
21 días previos del inicio de síntomas:	
Relación sexual sin protección	8
Viaje a zona endémica	0

Cuatro pacientes tenían diagnóstico previo de HIV. Todos presentaban recuento de CD4 mayor a 200 cél/ml y tres de ellos se encontraban en tratamiento con terapia antirretroviral (TARV). Ningún paciente presentó ITS concomitantes al momento del diagnóstico de viruela símica.

**Figura 1:**
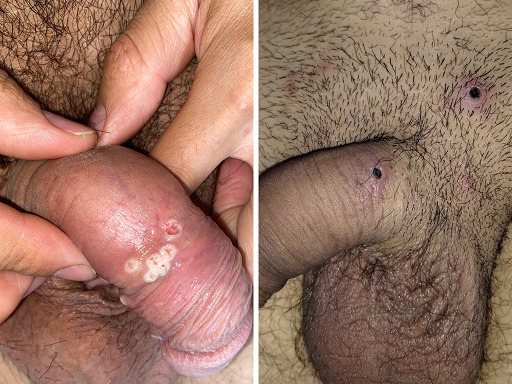
pseudopústulas en región genital masculina.

Las lesiones elementales encontradas en los exantemas fueron: pápulas (9/10), pústulas (8/10), costras (8/10), pseudopústulas (7/10), úlceras (3/10), vesículas (2/10) y máculas (1/10). Todos los pacientes tenían lesiones a nivel genital, perianal y/o glúteos (Figuras 1 y 2).

**Figura 2:**
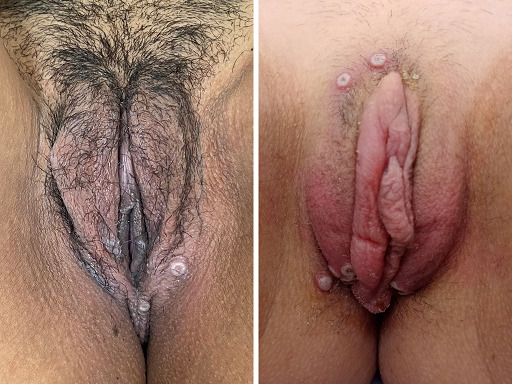
pseudopústulas en región genital femenina.

**Figura 3:**
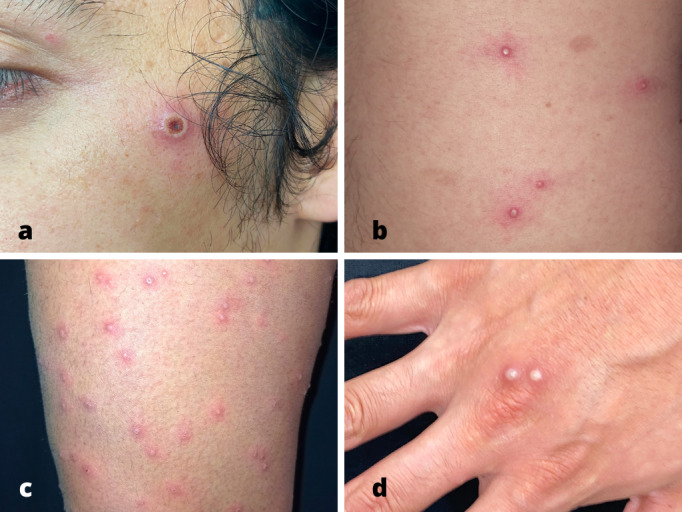
pseudopústulas en cara (a), tronco (b), miembro inferior (c) y dorso de mano (d).

Además, la localización en el tronco se presentó en el 90% de los casos, seguida de miembros superiores (70%), miembros inferiores (60%) y cabeza y cuello (60%) (Figura 3).

La mayoría de los pacientes (9/10) presentaba más de 5 lesiones y afectación de 2 o más localizaciones. Todos los casos presentaron signos y síntomas asociados: adenomegalias dolorosas (90%), astenia (80%) y fiebre (60%). Estos aparecieron antes del exantema en 4 pacientes, de manera concomitante en otros 4 y luego del brote cutáneo en los 2 restantes. Ningún paciente presentó complicaciones graves, sin embargo, se observó edema local (2/10), proctitis (2/10), conjuntivitis (1/10) y faringitis (1/10). Las características clínicas detalladas por paciente se encuentran en la tabla 2.

**Tabla 2:** características clínicas de los diez casos diagnosticados en el Servicio de Dermatología del HIGA "Gral. San Martín" de La Plata.

			Lesiones elementales							Localización				
Paciente	Sexo	Edad	Máculas	Pápulas	Vesículas	Pústulas	Pseudopústulas	Costras	Úlceras	Genital	Perianal	Glúteos	Otra localización	Número de lesiones
1	F	20	-	✓	-	✓	✓	✓	-	✓	-	-	✓	> 20
2	F	49	-	✓	-	✓	-	✓	-	-	-	✓	✓	> 20
3	F	25	-	-	-	✓	✓	-	-	✓	-	-	-	< 5
4	M	37	✓	✓	✓	✓	-	✓	-	✓	-	-	✓	entre 5 y 20
5	M	30	-	✓	-	✓	✓	✓	-	✓	-	✓	✓	entre 5 y 20
6	M	30	-	✓	-	✓	✓	-	✓	-	✓	-	✓	> 20
7	M	37	-	✓	-	✓	✓	✓	✓	-	✓	✓	✓	> 20
8	M	43	-	✓	✓	-	✓	✓	-	✓	-	-	✓	entre 5 y 20
9	M	51	-	✓	-	-	-	✓	-	✓	-	-	✓	entre 5 y 20
10	M	31	-	✓	-	✓	✓	✓	✓	✓	✓	✓	✓	> 20

## Discusión

La mpox es una infección zoonótica causada por el virus Monkeypox, un ortopoxvirus que pertenece al mismo género que el virus causante de la viruela. ^1-4^ Fue descripta en humanos por primera vez en 1970 en la República Democrática del Congo en personas no vacunadas contra la viruela. A partir de ese momento, se reportaron casos en otros países de África Central y Occidental donde es considerada una enfermedad endémica. Desde mayo del año 2022 se identificaron múltiples casos en países no endémicos. ^1-10^ Debido al rápido aumento de notificación de los mismos, la Organización Mundial de la Salud (OMS) declaró el brote como emergencia de salud pública de importancia internacional. ^3-6,8, 9^ Existen más de 87.000 casos reportados a la fecha que afectan a 111 países. ^11^ En Argentina se han informado 1.129 casos. ^12^

Debido al descenso de los casos a nivel mundial, en mayo del año 2023 finalizó la emergencia sanitaria. ^11^ En nuestro servicio el último caso registrado fue en noviembre del 2022. Esto podría deberse a un aumento de la inmunidad innata, la vacunación y cambios en el comportamiento de los grupos más vulnerables. Por otro lado, las medidas de salud pública que promueven la detección temprana y el aislamiento de los pacientes también fueron un factor importante. ^1 4^

La forma clásica de transmisión es a través del contacto con fluidos corporales (saliva, secreción respiratoria o de lesiones cutáneas), heces, mordeduras o ingesta de carne de animales

infectados. ^6^ El reservorio suelen ser roedores silvestres. ^1 4^ El virus también puede propagarse entre humanos mediante contacto directo, indirecto a través de fómites, secreciones respiratorias y transmisión vertical. ^1,7, 8^

Las características epidemiológicas han cambiado desde la aparición de los primeros casos. El contacto directo con la piel o mucosas con lesiones durante el acto sexual es el principal método de transmisión en el brote actual. Las microabrasiones en las mucosas podrían facilitar esta vía de contagio. ^1,7,8, 10^ A pesar de esto, mpox no debería considerarse una ITS ya que puede transmitirse de cualquier forma que implique contacto estrecho prolongado. ^1^ Si bien se ha detectado ADN viral en muestras de semen mediante PCR, estos hallazgos no son suficientes para comprobar la infectividad. ^4, 6-8^

La mpox afecta principalmente a hombres jóvenes que tienen sexo con hombres, al igual que lo observado en nuestra casuística. ^1,2,4,7-9, 13^ Sin embargo, puede afectar a cualquier persona con conductas sexuales de riesgo independientemente de su edad, género y orientación sexual. ^1,5, 6^

Es importante conocer esto ya que son más frecuentes los errores y retrasos diagnósticos de la enfermedad en mujeres cis y personas no binarias en comparación con mujeres trans y hombres. ^5 9^ Català et al. describen la enfermedad en 185 pacientes de España, donde el 100% eran hombres. ^2^ Por otro lado, en una serie de casos global, Mitjà et al. informan que el 96% de sus pacientes eran de sexo masculino. ^13^ En Argentina, el 98% de los pacientes con mpox fueron de sexo masculino. En nuestra muestra presentamos 3 mujeres cis de las 24 reportadas en nuestro país. ^12^ Sin embargo, consideramos que el número de casos es una limitante en nuestro estudio. La mayoría de nuestros pacientes tenía antecedentes de contacto sexual de riesgo. Ninguno presentó una ITS concomitante, a diferencia de lo informado en la literatura que lo describe en un 13 a 76%. ^1,2, 7-9^

La forma endémica comienza con síntomas prodrómicos como fiebre, cefalea, mialgias y astenia. El brote cutáneo aparece 1 a 3 días después del inicio de los síntomas generales y afecta principalmente cara y tronco para luego distribuirse al resto del cuerpo de manera centrífuga. En un primer momento se pueden observar máculas que posteriormente evolucionan a pápulas, vesículas, pústulas y costras. Las complicaciones más frecuentes son dolor, ulceración y sobreinfección bacteriana ^.(1,4,6,8,10)^

En el brote actual las lesiones suelen comenzar en la región anogenital, considerado el sitio de inoculación, con posibilidad de extenderse hacia el resto del cuerpo. ^1, 6-8^ La cantidad de lesiones cutáneas es variable y está descripta una media de cinco. ^1^ Los síntomas sistémicos son muy frecuentes y pueden aparecer antes, durante o después del brote cutáneo. ^1,6, 8^ Todos nuestros pacientes tenían afectación de la región anogenital o glútea, el momento de aparición de síntomas sistémicos fue variable y la mayoría presentó más de cinco lesiones. Además de las lesiones elementales anteriormente descriptas, los pacientes pueden presentar úlceras dolorosas y pseudopústulas (pápulas sólidas en las que es imposible destechar y obtener contenido purulento) con centro necrótico. ^1 2^ Català et al. fueron los primeros en describir estas lesiones en mpox y reportaron su aparición en un 75% de los casos. ^2^ En nuestra muestra se encontraron en un 70%. Están descriptas nuevas complicaciones como proctitis, faringoamigdalitis ulcerosa y edema genital, observadas también en nuestra serie de casos. ^1,7, 8^ El compromiso ocular es muy infrecuente, alrededor del 1% de los pacientes presentan esta complicación en el brote actual. En cambio, en la forma endémica puede aparecer en un 9 a 23% de los casos. Se suele manifestar como blefaritis o conjuntivitis. ^10^ Esta última se encontró en uno de nuestros pacientes.

Existen grupos vulnerables con riesgo de complicaciones más graves, como por ejemplo mujeres embarazadas, niños y pacientes con HIV. ^4 8^ Entre el 24 y 50% de los pacientes con mpox tenían diagnóstico previo de HIV con buen control de la enfermedad, lo que coincide con nuestra serie de casos. ^1,2,6-9, 13^ Las formas graves se describieron en individuos con recuento de CD4 menor a 200 cél/ml y se caracterizaron por necrosis cutánea masiva, afectación pulmonar y mayor severidad del compromiso oral y anogenital. ^3 13^ Todos nuestros pacientes presentaban recuento de CD4 mayor a 200 cél/ml y no se observaron diferencias en cuanto a las manifestaciones clínicas o complicaciones con respecto al resto de los casos.

Se debe sospechar mpox en aquellos pacientes que presenten antecedentes epidemiológicos y manifestaciones clínicas características. Es necesaria la confirmación mediante la detección de ADN viral con PCR en muestras de lesiones de piel o mucosas. ^1,6, 14^ Otras técnicas de diagnóstico incluyen la visualización del virus en microscopía electrónica, pruebas serológicas, histopatología cutánea y tinción con inmunohistoquímica. La anatomía patológica es similar a la de otros ortopoxvirus. En ella se pueden observar cuerpos de inclusión citoplasmáticos eosinofílicos, células gigantes multinucleadas, necrosis epidérmica, acantosis, degeneración balonizante, espongiosis e infiltrado inflamatorio perivascular. ^1,8,15, 16^

El diagnóstico diferencial debe realizarse con otras infecciones virales que puedan manifestarse con vesículas y pústulas como varicela, herpes zoster y herpes simple. ^1,8, 14^ El test de Tzanck presenta los mismos hallazgos en todas estas enfermedades, por lo que no es una herramienta útil para diferenciarlas. ^15^ Sin embargo, en la histopatología, los virus de la familia Herpesviridae provocan cuerpos de inclusión en el núcleo de los queratinocitos infectados, en lugar de encontrarse en el citoplasma como ocurre en los Orthopoxvirus. ^15^ Debido a la morfología de las lesiones en el brote actual y al compromiso anogenital de las mismas, también se deben tener en cuenta ITS como sífilis, linfogranuloma venéreo, chancro blando y molusco contagioso como parte de los diagnósticos diferenciales. ^1 14^

Los pacientes con viruela símica deben cumplir aislamiento de contacto y respiratorio hasta que todas las lesiones se encuentren reepitelizadas, momento en el cual se considera que finaliza el período de infección. En Argentina los tratamientos antivirales específicos, como tecovirimat, y la vacunación no están aprobadas. Por lo tanto, se realiza manejo sintomático de la enfermedad y se considera el uso de antivirales en protocolos de investigación en pacientes con síntomas graves o con alto riesgo de complicaciones.(1,3,14) Ninguno de nuestros pacientes presentó complicaciones graves.

## Conclusión

En nuestra muestra presentamos 3 pacientes de sexo femenino del total de las 24 mujeres reportadas en el país.

En la mayoría de los casos observamos pseudopústulas, lesión elemental descripta recientemente para esta entidad.

Uno de los pacientes de la casuística presentó compromiso ocular, complicación reportada en un 1% de los casos en el brote actual.
